# Basivertebral Nerve Ablation for Vertebrogenic Low Back Pain in an Elderly Patient With Multilevel Modic Changes: A Case Report

**DOI:** 10.7759/cureus.85792

**Published:** 2025-06-11

**Authors:** Shivang Patel, Victoria K Corder, Tom Sussan, Joachim Kavalakatt, Tony El-Hayek

**Affiliations:** 1 College of Medicine, Lake Erie College of Osteopathic Medicine, Bradenton, USA; 2 Pain Management, Mercy Health - Allen Hospital, Oberlin, USA

**Keywords:** basivertebral nerve ablation, chronic low back pain (clbp), minimally-invasive spine, modic changes, vertebrogenic pain

## Abstract

Chronic low back pain (CLBP) is a common disabling condition, especially in older patients with degenerative lumbar pathology. This case report involves an 84-year-old man with a history of diabetes mellitus (DM) and long-standing low back pain, mainly in the left lower lumbar area, unresponsive to conservative management. Imaging showed multilevel degenerative changes, including Modic type 1 and 2 changes, suggestive of vertebrogenic pain. As he did not respond to standard treatments, basivertebral nerve (BVN) ablation was proposed as a novel treatment modality. This case points to the importance of diagnosing vertebrogenic pain in chronic pain treatment and highlights BVN ablation as a novel treatment modality.

## Introduction

Chronic low back pain (CLBP), experienced by 13% of adults in the United States, is defined as the persistence of pain in the lumbar region for 12 weeks or longer [1]. This debilitating condition can cause medical and economic burdens, leading to a decrease in health and well-being. Individuals with CLBP can experience decreased physical function, increased limitations on social activities, increased depressive symptoms, and an overall lower health-related quality of life [2]. For geriatric populations, CLBP is the most common health problem, with a prevalence ranging from 36% to 70%. While there are many potential causes and risk factors for CLBP, the etiology is typically idiopathic [3]. For women, they are two times more likely to experience chronic CLBP compared to men at all ages.

For CLBP, more in-depth research has discovered a common cause being vertebrogenic back pain. Vertebrogenic back pain is defined as CLBP that originates from degeneration causing increased sensitivity to the basivertebral nerves (BVNs) and the endplates in which it innervates [4]. The BVN, a source of nociceptive fibers, enters the vertebra through the central vascular foramen and then migrates to innervate the inferior and superior endplates. As vertebral degeneration occurs, these fibers are seen to multiply and generate increased pain in this area [5]. Through varying studies, BVN ablation has been shown to be a novel treatment for vertebrogenic pain. BVN ablation is a minimally invasive procedure in which radiofrequency energy generates heat to target and disrupt the function of the BVN. This procedure is characteristically performed for patients who suffer from CLBP for more than six months and their pain is refractory to other treatments [6].

Here, we present the case of an 84-year-old male with refractory CLBP secondary to complex spinal pathology, who experienced significant pain relief following BVN ablation.

## Case presentation

We present a case of an 84-year-old male with a history of type 2 diabetes mellitus (DM) and CLBP for more than several years. The patient subjectively described his pain as deep, aching, and primarily at the left lower lumbar region. The pain was invariably triggered by seated flexion, long sitting, standing, walking, and bending - typical clinical characteristics suggestive of an anterior column-mediated vertebrogenic pain.

The patient was subjected to intensive conservative treatment, including pharmacotherapy with acetaminophen, neuropathic agents, central muscle relaxants, and opioids (currently tramadol 50 mg every six hours as needed). He also underwent a formal six-week program of physical therapy in the last six months, but improvement was compounded by intolerance and worsening during treatments. Interventional treatments like epidural steroid injections (ESIs), medial branch blocks (MBBs), and radiofrequency ablation (RFA) to the posterior elements were not effective in providing notable relief. Cognitive and behavioral elements of chronic pain were treated in conjunction with his primary care provider, and no significant psychosocial barriers were found. Despite this, the patient continued to report a visual analog scale pain score >6/10 with activity and extreme limitations in the activities of daily living, such as ambulation, transfer, and sleep.

The early 2025 lumbar spine magnetic resonance imaging (MRI) revealed evidence of multiple degenerative changes. Of note, Modic type 1 signal changes at L5-S1 on the anterior aspect of the inferior endplate of L5 - new compared with the previous imaging in 2023 - were suggestive of chronic inflammation. Modic type 2 changes at L3-L4 and L4-L5 were consistent with chronic fatty endplate degeneration. Other disclosures were diffuse desiccation of the discs, moderate stenosis of the central canal at L4-L5 with narrowing of the subarticular recess on both sides, bilateral foraminal stenosis, and severe left foraminal space of L5-S1. In the context of these radiographic findings, the pattern of pain, lack of radicular complaint, and nonspecific response to facet- or nerve root-directed modalities pointed most likely to discogenic vertebrogenic pain as the etiology.

**Figure 1:**
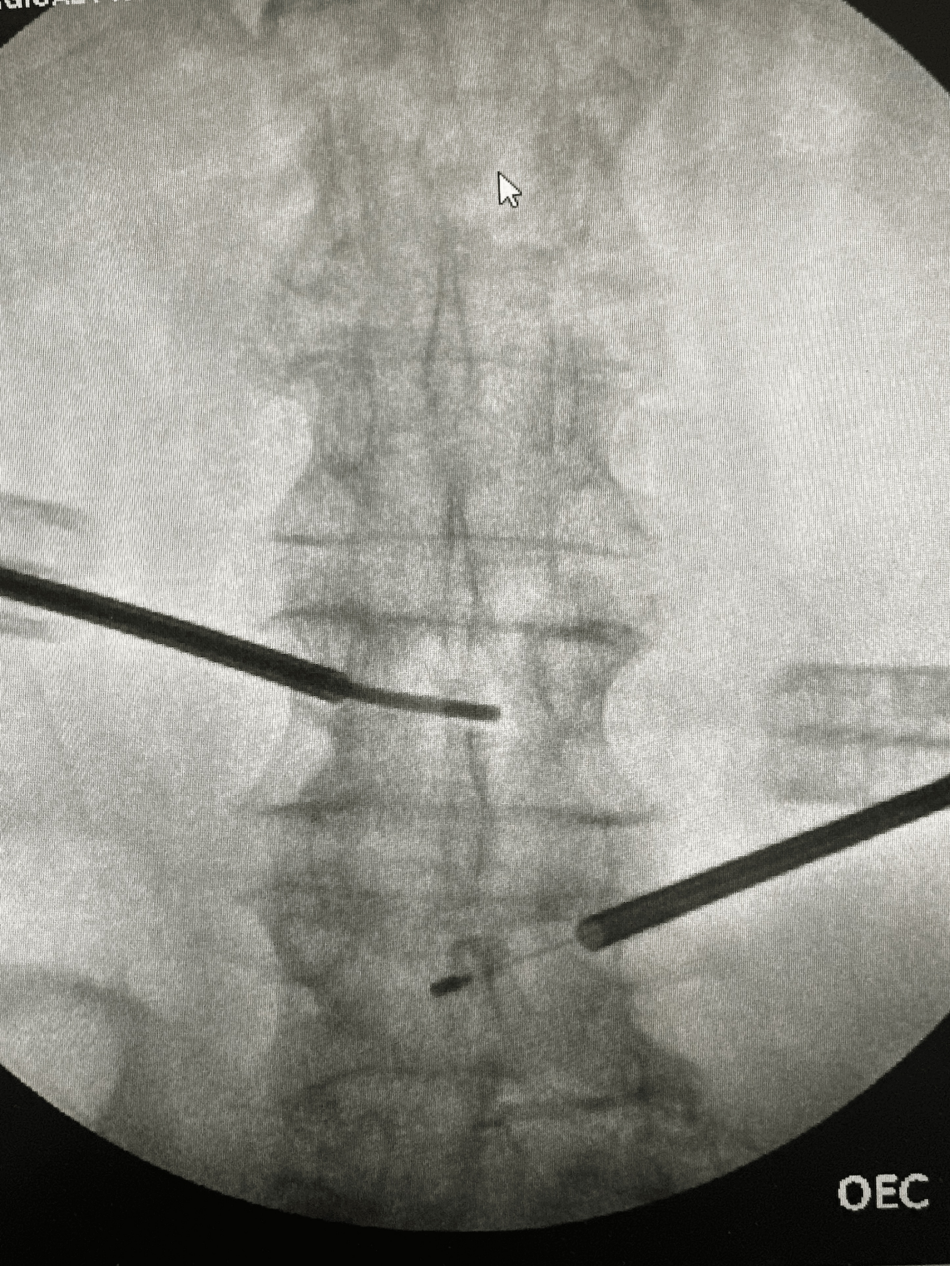
Fluoroscopic image showing transpedicular probe placement for basivertebral nerve ablation at the L3-S1 vertebral endplate with radiographic evidence of Modic endplate changes.

Based on the chronic nature of the symptoms, failure of traditional therapy, functional disability, and MRI findings in line with vertebrogenic pain, the patient was an ideal candidate for BVN ablation. The patient underwent multilevel BVN ablation at L3, L4, L5, and S1 under general anesthesia. After being positioned prone, the lumbosacral area was sterilized and draped. Each vertebral level was accessed through the pedicle using an 8-gauge cannula under fluoroscopic (X-ray) guidance (Figure 1). A curved delivery system with a flexible J-shaped wire was slowly advanced to the area inside the vertebral body where the BVN is located. A radiofrequency probe was then inserted at each level, and thermal ablation was performed at 85°C - lasting 15 minutes at S1 and seven minutes each at L5, L4, and L3 - using Relievant's standard radiofrequency algorithm. Once ablation was completed, all tools were removed, the small incisions were sealed with skin adhesive, and the patient was taken to the recovery area in stable condition with well-managed pain.

The procedure went smoothly. During the four-week follow-up, the patient reported decreased severity of pain and improved endurance for activities such as sitting, standing, and walking. Improvement in functional status and reduction of his analgesic medication consumption were noted in the three-month follow-up.

This case highlights the importance of BVN ablation as a treatment in patients with chronic vertebrogenic low back pain, particularly in older patients who have not responded to conservative and interventional therapies. The presence of Modic changes, particularly type 1 or 2 signal changes on MRI, is a critical imaging biomarker for the selection of candidates most likely to benefit from this treatment.

## Discussion

CLBP is a common condition, but in only around 20% of cases is it possible to define a certain patho-anatomical cause [7]. This diagnostic limitation emphasizes the need to stratify CLBP patients into clinically relevant subgroups, guided by underlying anatomical and pathophysiological features. One such subgroup includes patients exhibiting Modic changes, which are MRI-detected signal alterations in vertebral bone marrow adjacent to the endplates [7].

These changes are seen in 18-58% of the patients with CLBP and have been strongly associated with the condition [7]. Their presence has strong diagnostic and therapeutic implications. Modic changes are categorized into three types, namely type 1 (bone marrow edema and inflammatory changes), type 2 (conversion of red hematopoietic marrow to fatty marrow due to ischemia), and type 3 (subchondral bone sclerosis) [8]. In this patient, Modic type 1 changes were seen at L5-S1 and type 2 changes at L3-L4 and L4-L5, indicating a chronic degenerative process consistent with vertebrogenic pain. Emerging evidence suggests vertebral endplates play a pivotal role in CLBP pathogenesis. Due to their extensive blood supply and rich innervation - particularly from the BVN and venous plexus - these structures are highly susceptible to microfractures, inflammation, degeneration, and intraosseous fluid accumulation [6]. This anatomical vulnerability positions the endplates and their BVN innervation as key generators of axial spinal pain.

Studies have reported endplate damage in up to 43% of patients with chronic axial back pain [6]. Patients with vertebral endplate-related pain typically present with deep, aching lumbar pain exacerbated by standing, sitting, and spinal flexion - without radicular symptoms such as motor weakness or numbness [6]. This patient exhibited hallmark features of vertebrogenic pain, including deep lumbar aching exacerbated by seated flexion, standing, walking, and bending. While the literature supports BVN ablation in appropriately selected patients with Modic changes and treatment-refractory axial CLBP, this case is unique in several respects.

First, the patient’s pain was predominantly unilateral, localized to the left lower lumbar region. Vertebrogenic pain is traditionally characterized as bilateral and midline, and such unilateral presentations are uncommon. This atypical pattern introduces diagnostic ambiguity and can mislead clinicians toward facet, foraminal, or sacroiliac etiologies, causing vertebrogenic pain to be overlooked. Additionally, BVN ablation is primarily studied in patients with bilateral pain; successful treatment in this patient with unilateral symptoms expands the clinical profile of who may benefit.

Second, this case highlights the diagnostic challenge and therapeutic frustration associated with extensive treatment failure. The patient underwent a wide range of pharmacologic interventions - including acetaminophen, neuropathic agents, muscle relaxants, and opioids - as well as physical therapy and multiple interventional procedures (ESI, MBB, and RFA), all without meaningful improvement. In the context of continued pain, functional impairment, and imaging findings of Modic type 1 and 2 changes, BVN ablation emerged as a logical and ultimately successful next step.

Clinical studies to date have enrolled patients similar to our case: chronic axial LBP lasting >6 months, refractory to conservative therapy, and Modic type 1 or 2 changes on MRI from L3-S1 [9,10]. For example, the Intracept procedure, a minimally invasive BVN ablation technique, has been shown in randomized controlled trials to produce durable pain relief and functional improvement [9,11]. A five-year follow-up study demonstrated sustained improvement in Oswestry Disability Index (ODI) and pain scores, with no significant adverse events [12].

This case reinforces several important clinical considerations in the diagnosis and management of CLBP. Vertebrogenic pain, while common, is frequently underrecognized - particularly in older adults with complex degenerative changes and cases that deviate from the typical presentation patterns. In this patient, the pain was unilateral and localized to the left lower lumbar region, which is atypical for vertebrogenic pain, as it is most commonly midline or bilateral. This unusual presentation made diagnosis more challenging and underscored the importance of correlating subtle clinical findings with imaging evidence, such as the Modic type 1 and 2 changes observed here, to identify vertebral endplate pathology as the primary pain generator.

BVN ablation is a minimally invasive, targeted procedure that has shown strong evidence for treating vertebrogenic pain in appropriately selected patients. However, clinical trials have predominantly focused on patients with bilateral axial pain, and elderly individuals or those with atypical, unilateral symptoms are often excluded. This case demonstrates that patients with nontraditional presentations - like unilateral pain - can still experience significant improvements in pain, mobility, and medication dependence following BVN ablation. As such, this report supports expanding the clinical profile of BVN ablation candidates and reinforces the need for heightened diagnostic awareness when evaluating atypical presentations of CLBP.

## Conclusions

CLBP is a leading cause of disability in older adults, and accurate identification of the underlying pain generator is essential for effective management. This case draws attention to vertebrogenic pain - a commonly overlooked subtype of CLBP - identified by non-radicular, mechanically triggered axial symptoms and corresponding Modic type 1 and 2 changes on MRI. Notably, the patient presented with unilateral low back pain, which is atypical for vertebrogenic pain and can obscure the diagnosis. Despite being refractory to extensive conservative treatments, the patient experienced significant improvements in pain, function, and overall quality of life following BVN ablation. This case underscores the importance of recognizing unilateral pain patterns that may still reflect vertebrogenic origin and the value of Modic changes on MRI as critical biomarkers to guide targeted, minimally invasive interventions like BVN ablation in carefully selected patients.
